# Correlation between Ultrasound Reflection Intensity and Tumor Ablation Ratio of Late-Stage Pancreatic Carcinoma in HIFU Therapy: Dynamic Observation on Ultrasound Reflection Intensity

**DOI:** 10.1155/2013/852874

**Published:** 2013-12-26

**Authors:** Hui-Yu Ge, Li-Ying Miao, Jin-Rui Wang, Liu-Lin Xiong, Fang Yan, Cui-Shan Zheng, Jian-Wen Jia, Li-Gang Cui, Wen Chen

**Affiliations:** ^1^Department of Ultrasound, Peking University Third Hospital, 49 North Garden Road, Haidian District, Beijing 100191, China; ^2^Department of Urology, Peking University People's Hospital, No. 11 Xizhimen South Street, Xicheng District, Beijing 100044, China; ^3^Clinic Epidemiology Research Group, Beijing Anding Hospital, Capital Medical University, No. 5 Ankang Hutong, Xicheng District, Beijing 100088, China

## Abstract

The minimally invasive high-intensity focused ultrasound (HIFU) therapy is thermal ablation treatment for late-stage pancreatic carcinoma with widely recognized safety and effectiveness, but there are currently no instant assessment methods for its ablation effect. It is vital to find a real-time high-sensitive assessment method. This research aims to dynamically observe the variation rules of ultrasound reflection intensity, analyze the correlation between ultrasound reflection intensity and tumor ablation ratio, and find out the value of ultrasound reflection intensity in prognosis of HIFU ablation effect. HIFU intermittent therapies were retrospectively analyzed for 31 subjects with late-stage pancreatic carcinoma from March 2007 to December 2009 in the study. The variation rules of the ultrasound reflection intensity during HIFU therapy were summarized and the correlation between ultrasound reflection intensity and tumor ablation ratio was analyzed based on the tumor ablation ratio indicated by CT scanning. The conclusion is that variation of ultrasound reflection intensity can be used for initial assessment of tumor ablation in HIFU therapy and early prognosis of overall HIFU ablation, providing important clinical basis for improving safety and effectiveness of HIFU therapy. Ultrasound can work as a real-time imaging instrument for observation of HIFU ablation effect in treating late-stage pancreatic carcinoma.

## 1. Introduction 

With widely recognized safety and effectiveness in treating late-stage pancreatic carcinoma [1–4], the instant assessment of tumor ablation effect in the high-intensity focused ultrasound (HIFU) therapy has been barely researched in detail or reported. Therefore, it is important to find a real-time highly sensitive assessment method for HIFU ablation therapy. At present, 2D ultrasound is the main imaging method for tumor localization, real-time monitoring, and observation of the treatment effects in HIFU therapy. Some scholars [5–8] who have observed variations in the ultrasound reflection intensity within in vitro or in vivo animal experiments in the prognosis of HIFU ablation effect reported that ultrasound reflection intensity was significantly correlated with the actual organ damage, which has not been reported in any of the clinical studies. Nevertheless, some other scholars [9] believed that it could be subjective to use ultrasound reflection intensity for the prognosis of HIFU ablation effect, and some even argued that, with the coagulation necrosis occurring after the HIFU irradiation, sonogram could not monitor or display the real tumor [10–12]. This study was a clinical study and aimed to dynamically observe the variation rules of the ultrasound reflection intensity in HIFU therapy for the late-stage pancreatic carcinoma, to analyze the correlation between the ultrasound reflection intensity and tumor ablation ratio, and to find out the value of the ultrasound reflection intensity in the prognosis of HIFU ablation effect.

## 2. Materials and Methods

### 2.1. Study Design

The HIFU ablation therapies were retrospectively analyzed for 31 subjects with late-stage pancreatic carcinoma from March 2007 to December 2009. The study protocol was approved by the Ethics Committee of the Peking University People's Hospital (Beijing, China). All the patients provided written informed consent after being informed of the potential benefits and risks of the HIFU therapy.

### 2.2. Patients

The inclusion criteria included (1) late-stage pancreatic carcinoma confirmed by pathologic diagnosis (*n* = 13), CT images and magnetic resonance imaging (MRI) (*n* = 18), (2) a single tumor, (3) tumors that were considered unresectable or were otherwise considered ineligible for conventional pancreatectomy, gastric bypass, or other recommended conventional surgery, (4) discontinued conventional antitumor therapies due to ineffectiveness or poor tolerability ≥1 month prior to HIFU, (5) patients undergone CT-guided HIFU ablation, and (6) patients who had complete medical histories, including relevant CT and other imaging reports indicating tumor size and dimensions and TNM classification according to International Union Against Cancer (UICC) criteria [13]. The exclusion criteria included (1) poor physical condition indicating likelihood for poor toleration of HIFU therapy, (2) an estimated survival time of ≤3 months, or (3) absence of proper ultrasound channels for safe HIFU treatment, as determined by the investigators.

### 2.3. HIFU Therapy

Patients were instructed to fast for 6–8 hours before the HIFU therapy and were treated with simethicone (2 mL) to reduce the gastric gas several hours prior to the procedure. Patients were placed in the supine or right lateral decubitus position with the ribs covered with an acoustic protector. They were then treated with 1 h/day of intermittent accumulative longitudinal ultrasound therapy without anesthesia using an FEP-BY02 HIFU system (Beijing Yuande Bio-medical Engineering Co. Ltd., China) with a single concave, spherical focusing element real-time ultrasound localization and monitoring, as previously described [2]. The number of treatments was determined by the size of the tumor. The applied ultrasound had a frequency of 1.04 MHz, an oval focal point with a 3 mm diameter and 8 mm length, a pulse with an emission time (*T*1) of 150 ms, interval time (*T*2) of 150–300 ms, pulse width of 300–450 ms, and duty factor of 33.3–50.0%, and a single-point emission frequency of 60–80. The distance was 4 mm between the spots/lines and 6 mm between the layers. Acoustic energy per spot was 700–1200 J, approximately 1000–1200 J in most treatments. All HIFU treatments were administered with a margin of 0.5 cm from the tumor surface in order to prevent the pancreatic leakage and thermal damage to the adjacent organs and tissues. Ambulatory electrocardiograms (ECG) and blood pressure were monitored throughout each therapy session.

### 2.4. Ultrasound

Pancreatic tumors were measured by 2D ultrasound and Color Doppler Ultrasound with C2-5 convex array probe before and after each HIFU treatment in order to observe internal ultrasound reflection and blood flow distribution and to store local static and dynamic image. During the process of each HIFU treatment, ultrasound was used to observe the real-time tumor changes, to record the starting time for the increase of ultrasound reflection intensity and its duration, and to observe and store images with C2-5 convex array probe pressing on the abdominal wall surface.

The images were assessed independently by two senior radiologists with more than 15 years of experience, according to the following criteria. Compared with the ultrasound reflection from its surrounding tissues, target tumors with an obvious increase of ultrasound reflection intensity were classified into Group A. Target tumors without an increase in ultrasound reflection intensity were classified into Group B. Also, target tumors with a different assessment from the two independent radiologists were classified into Group B.

### 2.5. CT Scanning

All the patients were examined by CT (GE Light Speed 16 Slice CT, USA) one week prior to the initiation of HIFU therapy to determine the appropriate treatment location and margins and one month after the HIFU therapy for followup. CT scans were independently reviewed by two blinded senior radiologists with more than 15 years of experience and the post- and pre-HIFU CT scans were compared. Maximum left-right (*A*), anteroposterior (*B*), and superoinferior (*C*) tumor diameters were recorded and the treatment area was calculated according to the formula, where *a*/*b*/*c* = (*A*/*B*/*C* − 1 cm)/2, representing the radius of the ellipsoid as follows:

(1) $$\begin{array}{c} V=\frac{4}{3}\cdot abc\cdot \pi . \end{array}$$

The target skin distance (TSD) was also recorded based on the pre-HIFU CT scans and was defined as the maximum distance between the skin surface and the rear edge of the tumor.

### 2.6. Tumor Ablation Assessment

The extent of tumor ablation effects following HIFU was assessed by examining the expansion of nonenhancement regions of the CT images. The ablation zone was calculated according to the formula of the ellipsoid. The ratio (%) of the tumor ablation zone to the therapeutic target area was defined as the tumor ablation ratio. Patient outcomes were classified by nonenhancement area as the tumor ablation ratios of more than 30%. Nonenhancement areas of more than 30% were considered as indicative of an association between HIFU therapy and tumor ablation (positive tumor ablation effects), while the tumor ablation ratios of less than 30% were considered as no association (negative tumor ablation effects).

### 2.7. Safety Assessments

Incidence of HIFU-related adverse events (AEs), including burns, subcutaneous fat callus, abdominal pain, pancreatitis, peritonitis, jaundice, hemorrhage, vascular injury, gastrointestinal perforation, and intestinal necrosis, were recorded.

### 2.8. Statistical Methods

All data were analyzed with SPSS v.13.0 (SPSS Inc., Chicago, IL, USA). Data were recorded as means ± standard deviations (SD), median, or frequency values for descriptive statistics. Independent sample *t*-tests and chi-square tests were applied for the analysis of the quantitative and enumeration data. Mann-Whitney *U* test was applied for skewed data. Logistic regression was applied using TSD by CT scanning and variation of ultrasound reflection intensity as independent variables and tumor ablation effect as the dependent variable. *P* values of less than 0.05 were considered statistically significant (*P* < 0.05).

## 3. Results

### 3.1. Demographics and Clinical Conditions of the Study Group

A total of 31 subjects with late-stage pancreatic carcinomas consisted of 20 men and 11 women with an age range of 36–83 years (66.6 ± 11.4). There were 21 (67.7%) pancreatic body carcinomas, 7 (22.6%) pancreatic head carcinomas, and 3 (9.7%) pancreatic tail carcinomas. The Pre-HIFU maximum longitudinal and horizontal tumor dimension ranges were 3.0–9.3 cm and 1.8–8.7 cm (mean dimension of 5.1 × 4.1 cm). According to the TNM staging, there were 9 (29.0%) subjects in stage III and 22 (71.0%) subjects in stage IV. The TNM stage IV cases included 10 cases of hepatic metastasis (45.5%), 2 cases of pulmonary metastasis (9.1%), 7 cases of peritoneal metastasis (31.8%), and 3 cases of hepatic and peritoneal metastasis (13.6%). Patients were previously treated with chemotherapy (7/31, 22.6%) and radiotherapy (3/31, 9.7%) without any success (Table 1).

### 3.2. Characteristics of the HIFU Therapy

**Figure 1:**
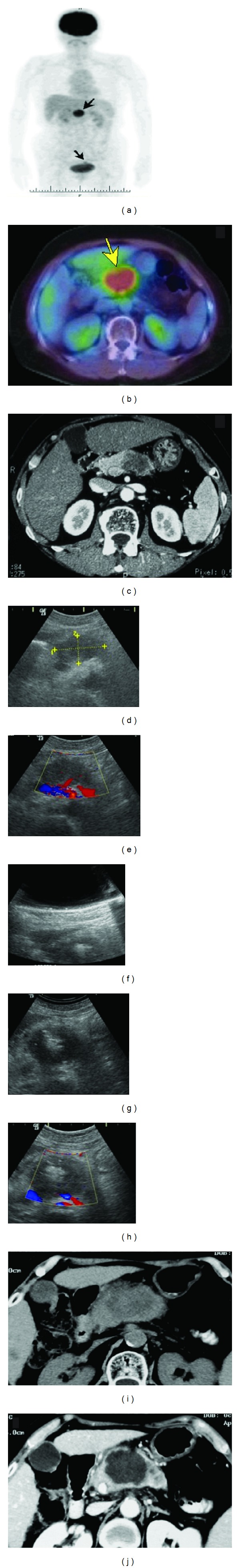
A 64-year-old woman with about 4.9 × 3.5 × 2.9 cm pancreatic body carcinoma. The pre-HIFU isotope scan (a) and PET-CT (b) showed a clear radioactive concentration in pancreas area. The enhanced CT (c) showed low-density lesions in pancreatic body with internal mild heterogeneous enhancement and marginal mild enhancement. Ultrasound showed hypoechoic lesions (d) with clear boundary, internal homogeneous ultrasound reflection, and small blood flow signals (e). In the 6th HIFU treatment, significant increase of ultrasound reflection intensity from tumor central area (f) was observed by ultrasound. Air-like hyperechoic ultrasound reflection (g) without blood flow signals (h) from inside of the tumor was observed by abdominal wall scanning. A month later, CT and enhanced CT showed tumor volume increase with internal uneven low density, no enhancement ((i), (j)), and tumor ablation ratio over 70%.

**Figure 2:**
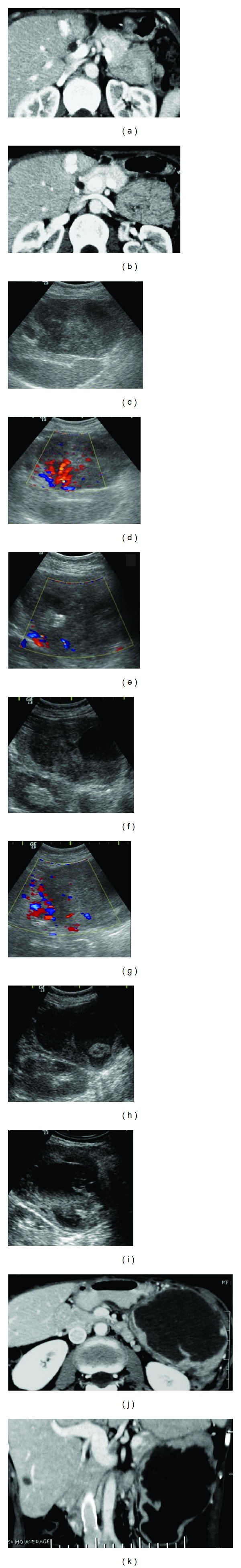
A 44-year-old woman with about 9.3 × 8.4 × 8.7 cm metastatic pancreatic tail carcinoma. The pre-HIFU enhanced CT (a) showed low-density lesions in pancreatic tail with clear boundary, internal heterogeneous echo, small liquefaction necrosis, and internal inhomogeneous enhancement (b). Ultrasound (c) showed a hypoechoic pancreatic tail with a clear boundary, internal heterogeneous ultrasound reflection, and oval anechoic area. The CDFI (d) showed a rich blood flow signal in tumor parenchyma. In the 5th treatment, ultrasound showed air-like hyperechoic reflection within tumor parenchyma; CDFI showed significant decrease of blood flow signal in tumor parenchyma (e). In the 8th treatment, liquefaction necrosis area increased, liquefied surface formed through necrotic material deposition (f), and blood flow signal decreased significantly in tumor parenchyma (g). In 12th (h) and 17th (i) treatments, tumor parenchyma decreased while liquefaction necrosis inside significantly increased with floating pellets or tablets of necrotic materials. A month later, the axial (j) and coronal (k) enhanced CT showed significant tumor volume increase with internal large liquefaction necrosis, a little marginal parenchyma and tumor ablation ration over 70%.

**Figure 3:**
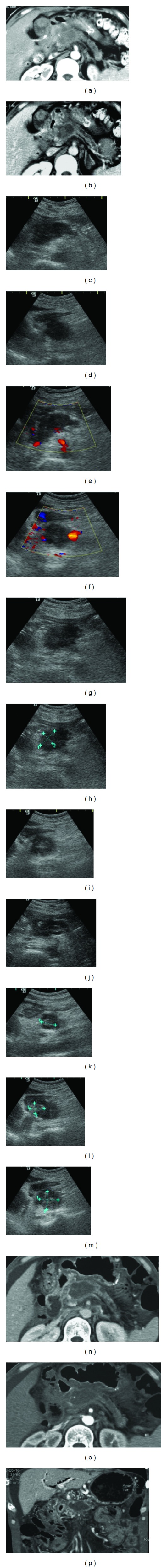
A 53-year-old man with about 5.0 × 2.6 × 2.2 cm pancreatic body carcinoma. The pre-HIFU enhanced CT showed uneven low-density lesions in pancreatic body with mild enhancement in parenchyma in arterial phase (a) and venous phase (b) and liquefaction necrosis. Ultrasound showed hypoechoic lesions ((c) cross section and (d) longitudinal section) with clear boundary, internal heterogeneous echo, and small blood flow signal from around the tumor ((e) cross section and (f) longitudinal section). In the 2nd treatment, ultrasound showed increase of ultrasound reflection intensity within 2.8 × 1.9 cm on the right lesions with unclear boundary ((g), (h)). In the 3rd treatment, ultrasound reflection intensity gradually increased with unclear boundary ((i) and (j)). In the 4th treatment, longitudinal ultrasound on the left lesions showed significant increase of ultrasound reflection intensity (k). In the 6th treatment, longitudinal ultrasonography showed increased reflection intensity in the upper area of the lesions (2.2 × 1.7 cm) (l). In the 8th treatment, ultrasound showed significant increase of ultrasound reflection intensity (m). A month later, enhanced CT ((n) arterial phase, (o) venous phase, and (p) coronal-section) showed no internal significant enhancement, but marginal ring enhancement and tumor ablation ratio over 70%. Patient ascites increased due to hypoalbuminemia.

**Figure 4:**
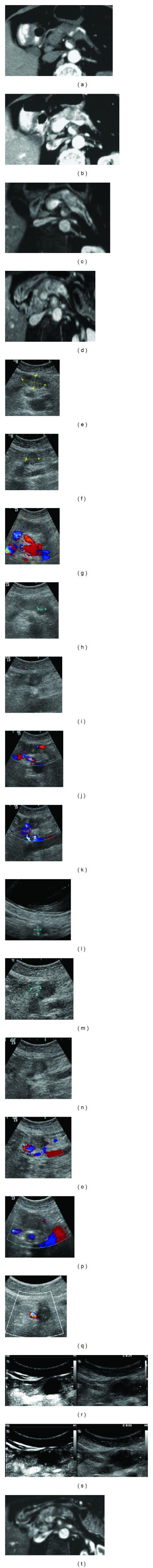
A 78-year-old woman with about 4.6 × 1.5 × 1.6 cm pancreatic body carcinoma. The pre- HIFU enhanced CT ((a) and (b)) and MRI ((c) and (d)) showed inhomogeneous enhancement within the lesions. Ultrasound showed hypoechoic lesions ((e) cross section and (f) longitudinal section) with clear boundary, internal homogeneous echo, and no obvious blood flow signal (g). In the 8th treatment, ultrasound showed increase of ultrasound reflection intensity within 1.0 × 1.0 cm ((h) cross section and (i) longitudinal section) on left lesions, but no obvious blood flow signal ((j) cross section and (k) longitudinal section). In the 12th treatment, ultrasound observed significant increase of ultrasound reflection intensity within 1.2 × 0.8 cm in the upper area of the lesions ((l) and (m)). In the 19th treatment, ultrasound showed mild enhancement in tumor central area with unclear boundary (n); CDFI showed no obvious internal blood flow signal (o). In the 20th treatment, ultrasound showed internal heterogeneous enhancement with unclear boundary and no blood flow signal ((p) cross section and (q) longitudinal section). After the whole HIFU treatments, ultrasound showed no contrast agent filling within the lesions ((r) 21′′ and (s) 55′′). A month later, enhanced MRI showed no obvious enhancement with the tumor central area (t).

The therapy frequency (number of HIFU treatments per patient) was 2–20 (mean: 9.5 ± 4.2). The post-HIFU tumor volume was 5.29–355.85 cm^3^ (mean: 60.04 ± 82.22 cm^3^). The average ablation time (HIFU duration time/number of HIFU treatment for each patient) was 36.8–74.7 min with a mean of 59.6 ± 8.5 min. The therapy duration was 131.0–1430.0 min with a mean of 576.9 ± 298.2 min. The pre-HIFU TSD by CT scanning was 4.5–11.5 cm with a mean of 8.1 ± 1.7 cm. Of the 31 patients, 17 (54.8%) exhibited a negative tumor ablation effect (tumor ablation ratio ≤ 30%); however, positive tumor ablation effects were also observed among 14 (45.2%) of the patients (tumor ablation ratio > 30%). The tumor ablation ratio was over 70% in only five (16.1%) patients. According to the variation of the ultrasound reflection intensity during the HIFU treatment, 14 (45.2%) subjects were classified into Group A, including 11 (78.6%) pancreatic body carcinomas, 2 (14.3%) pancreatic head carcinomas, and 1 (7.1%) pancreatic tail carcinoma, and 17 (54.8%) subjects were classified into Group B, including 10 (58.8%) pancreatic body carcinomas, 5 (29.4%) pancreatic head carcinomas, and 2 (11.8%) pancreatic tail carcinomas. In group A, a total of 139 treatment sessions were given (mean 10.2 ± 4.3 sessions) with an average time of 62.8 ± 6.9 minutes. Of those, in 92 cases (66.2%) the variation of the ultrasound reflection intensity was significantly observed. The first increase of the ultrasound reflection intensity occurred during the 2nd to 7th treatment session, with an occurrence ratio of 1/14 (7.1%), 2/14 (14.3%), 5/14 (35.7%), 2/14 (14.3%), 3/14 (21.4%), and 1/14 (7.1%), respectively. The increase of the ultrasound reflection intensity started at 30–40 min of the therapy (68/92, 73.9%). With the ongoing treatment, the ultrasound reflection intensity gradually increased to an air-like hyperechoic maximum level (3/14, 21.4%; 10/92, 10.9%). After the treatment, the ultrasound reflection intensity gradually decreased to normal level after 1-2 hours. In the three subjects with air-like hyperechoic ultrasound reflection, it decreased to a hypoechoic level. Additionally, with an increase of the treatment, the starting time of the ultrasound reflection intensity gradually advanced and was increased to different degrees (Figures 1, 2, 3, and 4).

### 3.3. Correlations between HIFU and Clinical Variables

Significant difference was found in TSD, TSD classification, and tumor ablation ratio between Groups A and B (*P* = 0.001, 0.002, < 0.001, resp.). No significant differences were observed in gender, age, tumor location, clinical stages, tumor size, therapy frequency, average tumor ablation time, or therapy duration between Groups A and B (Tables 2 and 3). A good negative correlation was found between the variation of ultrasound reflection and TSD (*r* = −0.547, *P* = 0.001). Likewise, a good positive correlation was found between the variation of ultrasound reflection and tumor ablation ratio (*r* = 0.739, *P* = 0.001). The sensitivity of the variation of ultrasound reflection intensity was 85.7% in the prognosis of tumor ablation ratio while its specificity was 88.2%. The concordance rate between the two radiologists' assessment was 90.3% (28/31) for the tumor ultrasound reflection intensity and the difference was insignificant (*P* > 0.05). Furthermore, no significant difference was observed between the results provided by the two independent radiologists for the mass ablation assessment (*P* > 0.05).

### 3.4. Safety Outcomes

In this study, mild abdominal pain and local abdominal tenderness were observed in 7 (22.6%) patients and subcutaneous fat callus was observed in 6 (19.3%) patients. The 2nd-degree superficial skin burn was observed in 4 (12.9%) patients, necessitating cessation of the HIFU therapy for a day. Only 1 (3.2%) patient exhibited pancreatic effusion by ultrasound examination, with a maximum depth of 1.6 cm on the 6th day during HIFU with abdominal dull pain for several days without an elevation of serum or urine amylase. As indicated by CT scanning, pancreatic effusion disappeared in a month after HIFU without any other intervention. No severe AEs or deaths were reported.

## 4. Discussion 

Instant assessment of the HIFU ablation effects could play an important role in understanding the dose-effect relationship between the therapeutic dose and the ablation effect as well as in the HIFU therapy safety and effectiveness improvement.

The effect assessment by comparing pre-HIFU and post-HIFU enhanced CT and/or MRI changes showed that they could not completely meet the clinical needs, in spite of a high image resolution. This could be because it was not real-time and could not detect the ablation effects at any time point during the treatment. In contrast, ultrasound was shown to remain the main method for the real-time monitoring and instant assessment of the ablation effects during the HIFU therapy process due to being real-time, inexpensive, and nonradioactive with a relatively high image resolution [14].

The instant assessment of HIFU ablation effects was conducted by observing the variation of the ultrasound reflection intensities from the tumor through the use of ultrasound [12, 15–17]. According to prior reports, the HIFU therapy could increase the ultrasound reflection intensity because of the interface increase during the process, cavitation, and tissue fluid boiling due to tissue coagulation necrosis, which in return helped in the visualization of the HIFU damages [11, 18–21]. Some scholars studied the correlation between the variations of the ultrasound reflection intensities and HIFU ablation effects in the in vitro or in vivo animal experiments. The results of these studies were not in line with each other and the actual conclusion is still in debate. Some scholars believed that the ultrasound reflection intensity increase could reflect the coagulation necrosis in the target tissue and had the potential to be as an important real-time indicator of the therapeutic dose [9]. Some others argued that with the coagulation necrosis after HIFU irradiation, sonogram may not necessarily display the real tumor [11]. Additionally, all the ultrasonographies might not be 100% reflective of the reality since during the treatment process, the probe has to be retreated to the wave source for the examination in order to avoid the blockage of the ultrasound propagation path, which could result in a lower image resolution, increased artifacts, and an unclear lesion display [9]. Therefore, some argued that it could be subjective to use the ultrasound reflection intensity for the HIFU ablation effect prognosis [9, 14].

This study identified the objective existence of an increased ultrasound reflection intensity after the HIFU therapy and the potential relation between the ultrasound reflection intensity variation and the ablation effect by dynamically observing the variation of the ultrasound reflection intensity during the HIFU process. Although the assessment was subjective and the variation of the ultrasound reflection intensity was not quantitatively evaluated, the concordance rate between the two radiologists' assessments was so high, and variation of the ultrasound reflection showed a good positive correlation with the tumor ablation ratio. The sensitivity and specificity of the ultrasound reflection intensity variation in the prognosis of tumor ablation ratio were also high. Therefore, when there was no objective, real-time, yet effective assessment method during the HIFU process, it could be beneficial to use the non-invasive real-time ultrasound reflection intensity variation for the instant assessment of the ablation effects.

The limitation of the conventional ultrasound in the assessment of HIFU ablation effects lied in the application of the positioned phased array probe. In order to avoid the ultrasound blockage of the propagation path, a small positioned probe with a higher frequency, poorer penetration, and a lower image resolution as compared to the convex array probe was chosen. The phased array probe was retracted to the wave source away from the body surface for ultrasound observation, which had to be through the degassed water. The higher frequency and longer distance resulted in a lower image resolution and more artifacts, which affected an accurate judgment of the ultrasound reflection intensity variation. In order to enhance the probe's penetration and to reduce the artifact's interference, the C2-5 convex array probe was applied. Images with a higher resolution were acquired with the C2-5 convex array probe, pressing on the abdominal wall surface, which helped to improve the display rate of the ultrasound reflection intensity. The stored images were independently assessed by two senior radiologists in order to reduce the false positive rate.

In this study, a significant difference was found in the TSD and TSD classification between Groups A and B. Likewise, a good negative correlation was found between the ultrasound reflection variation and TSD, which showed a correlation between the ultrasound reflection intensity increase and the tumor depth. The depth of tumor was less deep and the post-HIFU ultrasound reflection intensity variation was more obvious. A significant difference was found in the tumor ablation ratio between Groups A and B. Likewise, a good positive correlation was found between the ultrasound reflection variation and the tumor ablation ratio. The sensitivity and specificity of the ultrasound reflection intensity variation were both high for the prognosis of the tumor ablation ratio, which showed that the ultrasound reflection intensity could potentially serve as an initial observation method for tumor ablation effect. When the variation was more obvious, the tumor ablation ratio was larger, showing a better ablation effect. For example, in the three subjects with an air-like hyperechoic phenomenon, the ablation ratios were over 70% after the whole HIFU therapy. In the two subjects with an insignificant ultrasound variation, the ablation ratios were over 30% after the whole HIFU therapy, which showed the HIFU-related ablation. This was probably related to a target temperature of less than 65°C [17]. In the two subjects with significant ultrasound variations, the ablation ratios were below 30% after the whole HIFU therapy, which was believed to be related to the ultrasonic artifacts, such as reverb after the two radiologists' review.

The safety and effectiveness of the HIFU therapy in treating pancreatic carcinoma has been reported by our previous study [2], which was consistent with the results of this study. In our previous study, complications included superficial skin burns (3.4%), subcutaneous fat sclerosis (6.7%), and an asymptomatic pancreatic pseudocyst (1.1%). There were no severe complications or adverse events related to high intensity focused ultrasound therapy seen in any of the patients treated in both studies. Although some researchers thought that tumors might not be detected by ultrasound due to its coagulation necrosis [10, 21], which could result in an excessive therapeutic dose, long duration of treatment, further increase in the complications, and safety reduction [10–12], no adverse event occurred in this study. Therefore, we believe that HIFU treatment of the pancreas appears to be safe when the device was operated properly and appropriate protective measures had been taken during the treatment process. This observation demonstrated the safety of the HIFU therapy in treating pancreatic carcinoma, which may be more linked with the protective measures during the treatment process. These measures included the pretreatment fasting, simethicone intake to reduce gas in the gastrointestinal tract, attachment of sound absorption foil to ribs area, bladder filling with cold water, pressing force of the bladder on the stomach and the transverse colon above the pancreatic surface away, hand-testing temperature, and constant application of the coupling agent to protect the ultrasound path. Additionally, the range of the accurate positioning, real-time monitoring, and ultrasound treatment was at 0.5 cm from the inner edge, avoiding tissues damages in the vital structures, such as blood vessels and intestines, and pancreatic leakage. Preoperative patient education and postoperative antiscald drugs can help to reduce the incidence of complications.

As the first clinical research in the instant assessment of the HIFU ablation effect, the assessment was subjective and the variation of the ultrasound reflection intensity was not quantitatively evaluated. In order to reduce the incidence of the false positives, the images were independently assessed by two senior radiologists, and the subjects with different assessments were classified into the negative group. In addition, this study was conducted with a small number of subjects and was not a large randomized controlled study. Furthermore, an accurate observation of the ultrasound reflection intensity variation greatly relied on the image identification ability and operating skills of the radiologists.

Besides, this study could have adopted quantitative ways with more advanced techniques, such as elasticity imaging, to assess the ablation effect for the sake of higher sensitivity and specificity. According to the researches, the tissue elasticity properties could greatly change after the HIFU irradiation [22] and elasticity imaging could be applied to image monitoring and damage assessment in the HIFU therapy [22–24]. Among various kinds of elastic evaluation technologies, ultrasound elasticity imaging is a promising technology and has many distinct advantages, because it is a simple, quick, feasible, inexpensive, and noninvasive method and can be real-time dynamic evaluation for HIFU therapy. At present, there have been no published reports on the application of ultrasound elasticity imaging for the diagnosis of pancreatic carcinoma despite its wide application in the superficial tissue or liver lesions [25–28]. However, based on our findings in this study, ultrasound elasticity imaging might have a positive role in the assessment of the superficial pancreatic carcinoma ablation. However, it might have some limitations in its application for the deep pancreatic carcinoma because of ultrasonic attenuation characteristics. MR elasticity imaging is of course a perfect image modality for assessing the ablation effect qualitatively because MRI has unique capabilities for planning, guiding, and monitoring ablation treatments due to its inherent soft tissue contrast and many specialized imaging sequences [29]. However, there was no report about its application in human beings and the cost is expensive. In all, elasticity imaging is a promising image modality in assessing the instant ablation effect and further studies are needed.

## 5. Conclusion

Variation of the ultrasound reflection intensity could be used for the initial assessment of the tumor ablation in HIFU therapy and early prognosis of overall HIFU ablation, providing important clinical basis for improving safety and effectiveness of the HIFU therapy. Ultrasound could work as a real-time imaging instrument for the observation of HIFU ablation effects in treating late-stage pancreatic carcinoma.

## Figures and Tables

**Table 1:** Demographics and clinical condition of the patients.

Parameters	Study group (*N* = 31)
Gender (M : F)	20 : 11
Age (yr), mean ± SD (range)	66.6 ± 11.4 (36–83)
Pre-HIFU maximum tumor horizontal dimensions (cm), mean (range)	4.1 (1.8–8.7)
Pre-HIFU maximum tumor longitudinal dimensions (cm), mean (range)	5.1 (3.0–9.3)
TNM stage, *n* (%)	
III	9 (29%)
IV	22 (71%)
Metastasis in TNM stage IV patients, *n* (%) where *n* = 22	
Hepatic	10 (45.5%)
Pulmonary	2 (9.1%)
Peritoneal	7 (31.8%)
Hepatic and peritoneal	3 (13.6%)
Previous treatments, *n* (%)	
Chemotherapy	7 (22.6%)
Radiotherapy	3 (9.7%)

**Table 2:** Impact of the variables on variation of ultrasound reflection intensity.

Parameter	Variation of ultrasound reflection intensity		*T*-value/*Z*-value	*P* value
	Group A (significant) (*n* = 14)	Group B (insignificant) (*n* = 17)		
Age	63.1 ± 13.4	69.4 ± 9.4	1.547	0.133
Post-HIFU tumor size (cm^3^)	51.0 ± 89.8	67.5 ± 80.1	−0.953*	0.341
Therapy frequency	10.2 ± 4.3	8.9 ± 4.3	−0.823	0.417
Average tumor ablation time (min)	62.8 ± 6.9	57.0 ± 9.2	−1.961	0.059
Therapy duration (min)	649.3 ± 316.2	517.3 ± 287.4	−1.310*	0.190
TSD (cm)	7.1 ± 1.7	9.0 ± 1.3	3.516	0.001

*Mann-Whitney *U* test was applied for skewed data.

**Table 3:** The clinical condition and ultrasound grouping for 31 pancreatic cancers.

Characteristics	Group A (*n* = 14)	Group B (*n* = 17)	*χ* ^2^ */P *
Gender			
Male	6 (42.9%)	14 (82.4%)	3.648/0.056
Female	8 (57.1%)	3 (17.6%)	
Age			
≤60	5 (35.7%)	2 (11.8%)	2.634/0.268
61–75	6 (42.9%)	11 (64.7%)	
≥76	3 (21.4%)	4 (23.5%)	
Tumor location			
Pancreatic head	2 (14.3%)	5 (29.4%)	1.389/0.499
Pancreatic body	11 (78.6%)	10 (58.8%)	
Pancreatic tail	1 (7.1%)	2 (11.8%)	
Clinical stages			
Stage III	5 (35.7%)	4 (23.5%)	0.120/0.729
Stage IV	9 (64.3%)	13 (76.5%)	
Classification by TSD			
≤7 cm	9 (64.3%)	1 (5.9%)	9.460/0.002
>7 cm	5 (35.7%)	16 (94.1%)	
Classification by tumor ablation ratio			
≤30%	2 (14.3%)	15 (88.2%)	16.952/<0.001
>30%	12 (85.7%)	2 (11.8%)	
